# Analyzing the Effects of Nano-Titanium Dioxide and Nano-Zinc Oxide Nanoparticles on the Mechanical and Durability Properties of Self-Cleaning Concrete

**DOI:** 10.3390/ma16216909

**Published:** 2023-10-27

**Authors:** Fatma El-Zahraa M. Mostafa, Piotr Smarzewski, Ghada M. Abd El Hafez, Ahmed A. Farghali, Wafaa M. Morsi, Ahmed S. Faried, Taher A. Tawfik

**Affiliations:** 1 Civil Engineering Department, Faculty of Engineering, Fayoum University, Fayoum 2933110, Egypt; asg00@fayoum.edu.eg; 2 Faculty of Civil Engineering and Geodesy, Military University of Technology, 2 gen. Sylwestra Kaliskiego, 00-908 Warsaw, Poland; 3 Chemistry Department, Faculty of Science, Fayoum University, Fayoum 2933110, Egypt; gma03@fayoum.edu.eg; 4 Materials Science and Nanotechnology Department, Faculty of Postgraduate Studies for Advanced Sciences, Beni-Suef University, Beni Suef 2722165, Egypt; ahmedfarghali74@yahoo.com; 5 Building Physics Research Institute, Housing & Building National Research Center (HBRC), Cairo 11511, Egypt; dr.wafaam@yahoo.com; 6 Department of Construction and Building Engineering, High Institute of Engineering, 6th October City 3226010, Egypt; dr.taher.tawfik@gmail.com; 7 Institute of Construction and Architecture, Slovak Academy of Sciences, Dúbravská Cesta 9, SK-845 03 Bratislava, Slovakia

**Keywords:** self-cleaning concrete, nano-titanium dioxide, nano-zinc oxide, photocatalytic tests, corrosion rate, mechanical properties

## Abstract

The goal of this paper is to investigate the impact of nano-materials on the mechanical and electrochemical properties of self-cleaning concrete. Nano-titanium dioxide and nano-zinc oxide were used as additives for this purpose. Additionally, a comparative study on the effect of using these materials on the self-cleaning concrete’s characteristics was conducted. The dosages of nano-titanium dioxide (nps-TiO_2_) and nano-zinc oxide (nps-ZnO) used were 0, 0.5, 1, 1.5, 2, and 2.5% and 0, 1, 2, and 3% of the weight of the cement, respectively. The results showed that the optimum compressive strength and the lowest corrosion rate were fulfilled at 2.5% of nps-TiO_2_ and 1% of nps-ZnO, and using 2.5% of nps-TiO_2_ achieved the highest improvement in the corrosion rate. However, 1% for nps-TiO_2_ mixtures and 1% for nps-ZnO mixtures were the best ratios for flexural strength. On the other hand, for the corrosion rate, the samples were tested at 2 and 6 months. When nps-TiO_2_ and nps-ZnO samples were compared to the control sample, 2.5% and 1% of nps-TiO_2_ and nps-ZnO, respectively, showed the largest improvement in resistance to corrosion. Also, the self-cleaning property of the samples containing nano-materials (nps-TiO_2_ and nps-ZnO) was tested. As the results illustrated, the self-cleaning property of the samples was increased over time due to photocatalytic degradation. Furthermore, the results of the photocatalytic tests showed that nps-TiO_2_ samples outperformed nps-ZnO samples overall.

## 1. Introduction

Global population growth has brought new challenges for humanity, such as the high number of pollutants released as a result of the burning of fossil fuels. These toxic substances not only pose a threat to the environment and human health but they also reduce a concrete structure’s lifetime through acidification and deterioration, raising the expense of upkeep and restoration. One innovative strategy for improving air quality is the use of photocatalytic materials in urban construction elements [1]. The use of photocatalytic materials is a technique that has the potential to help decrease air pollution by using these materials in concrete (called “self-cleaning concrete”) [2]. The photocatalytic technique is not unique; it has been used since the 1960s [3]. Although the main focus has been concentrated on water treatment technologies, the use of photocatalytic oxidation in building materials has gained the attention of researchers since the 1990s [4]. Self-cleaning concrete automatically cleans its own surface [5]. Nature is the source of this self-cleaning technology [5]. Lotus leaf nanoparticles—which possess a superhydrophobic function and whose remains are carried away by rain, thus removing dirt and debris—are the inspiration for the surface of self-cleaning surfaces. This is a result of the photocatalytic activities [5]. Photocatalytic materials (such as TiO_2_ and ZnO) use energy from sunlight (or any other ultraviolet light sources) to oxidize organic compounds (such as nitrogen oxides (NOx), sulfur oxides (SOx), tobacco smoke, bacteria, etc.) and convert them to less harmful substances [2,5].

(1) $$Photocatalyticprocess\to NO_{X}+Ultravioltlightsource+photocatalyticmaterials$$

As a result of solar UV radiation absorption, photo-catalytic materials produce hydroxyl radicals and superoxide anions, which interact with pollutants like NOx to transform them into less hazardous molecules (this might be helpful in regions with significant levels of air pollution) [5]. Following this process, water is largely employed as a cleaning agent. Following a rainstorm, the building becomes clean and pollutant-free. Water droplets sweep away NOx dust [2,5]. The overall mechanism of photocatalysis can be represented generically as photochemical processes, as shown in Equations (2)–(7) [6,7].

(2) $$TiO_{2}\overset{hv}{\to }h^{+}+e^{-}$$

(3) $$e^{-}+O_{2}\to O_{2}^{-}$$

(4) $$h^{+}+OH^{-}\to OH^{*}$$

(5) $$H^{+}+O_{2}^{-}\to HO_{2}^{*}$$

(6) $$NO+HO_{2}^{*}\to NO_{2}+OH^{*}$$

(7) $$NO_{2}+OH^{*}\to HNO_{3}$$

Photocatalytic systems offer two key advantages: they keep the surface clean and ensure a cleaner environment. Photocatalytic materials are not only unique in that they have a wide range of uses, but they also provide numerous benefits, such as reduced maintenance costs and time (cleaning process); they also provide energy savings by lowering the temperature of, and thus cooling, the buildings [5]. Nano-materials have become one of the most widely used methods in recent years for creating innovative cement composites. In addition to improving the material’s mechanical performance, adding nanoparticles to cements gives them additional qualities, like self-cleaning, electrical conductivity, self-healing ability, porosity, and greater durability [8,9,10]. Nano-TiO_2_ is currently the most extensively utilized photocatalytic material because of its ability to block UV rays, remove impurities, and photolyze organic contaminants, as well as its benefits of high oxidation and reduction, safe, high durability, and renewable recycling [11,12,13,14]. Building materials may be able to perform photocatalysis and self-cleaning to a greater extent when combined with nano-TiO_2_ and cement-based materials [15,16,17,18].

Because of its nucleation impact, nano-TiO_2_ is also applied to cement-based materials in order to enhance their mechanical and hydration characteristics [19,20,21]. The addition of nano-TiO_2_ particles to the cement decreases the overall porosity of the hardened concrete mixture while improving the pore structure [22]. Also, the addition of nano-TiO_2_ can increase the development of C-S-H, which improves the performance of the flexural concrete [23]. In comparison to normal concrete, self-cleaning concrete has six times more surface area exposed to sunlight due to its high porosity and specific surface area, which increases the amount of light that nano-TiO_2_ can absorb [24].

According to the majority of the published research, ZnO nanoparticles are mainly used for self-cleaning purposes in cement mortars because of their photocatalytic capabilities that are similar to those of TiO_2_ [25,26,27]; ZnO is usually thought of as a TiO_2_ replacement since it can absorb a larger portion of the solar spectrum’s energy and lighter quanta [28]. Additionally, it has been demonstrated that adding ZnO to reinforced concrete can help lower the corrosion rate of implanted steel bars [29].

The mechanical and structural properties are susceptible to direct corrosion effects. The overall reaction of the building may also be impacted. According to scientific terminology, concrete corrosion is the progressive deterioration of metal via chemical, electrochemical, and electrolytic interactions within its environment. It usually develops as the concrete ages. It becomes a major issue when materials are lost to corrosion. The most typical instance of corrosion is the rusting of iron when it is subjected to environmental factors. Hydrated ferric oxide builds up on the surface and causes rusting.

To face this problem, recently, many researchers have used ZnO to reduce the corrosion. According to reports, the use of nps-ZnO in cement mortars and concrete has minimized environmental problems linked to the caliber of building materials, waste reduction, greenhouse gas emissions, and decreased expenses associated with manufacturing and maintenance [30]. It has been reported that the use of ZnO increases the reactivity of the extra cementitious components by speeding up cement hydration and releasing more heat [31]. Additionally, the use of ZnO reduced the rate of the corrosion of steel bars used in concrete [29].

Furthermore, earlier studies have indicated that the existence of voids in concrete causes a reduction in compressive strength [5]. Thus, the strength and durability, as well as the thermal, mechanical, and electrical characteristics, of cementitious materials would all be improved because the nanoparticle photocatalyst would fill the spaces between cement particles [5].

It should be emphasized that there are very few studies on the use of ZnO in conjunction with Portland cement for structural composite applications, particularly those examining the impacts and characterizations of this nanomaterial as a cement-based construction material. Additionally, there was a dearth of data on the electrochemical characteristics (resistance to corrosion rate) and characterization of utilizing TiO_2_ and ZnO nanoparticles in the mixing of concrete. Thus, the present study emphasizes the significance of including these nanoparticles in concrete mixing in order to have a significant influence on concrete sustainability. 

In light of the previously mentioned characteristics of nps-ZnO and nps-TiO_2_, the primary aim of this study was to examine the impact of TiO_2_ and ZnO nanoparticles on mechanical properties (compressive and flexural strength) and durability properties (self-cleaning efficiency). In addition, we wanted to examine the corrosion-resistant performance of the cementitious composite in different conditions, such as in tap water and saline water. To this end, the Tafel potentiodynamic polarization technique was employed in order to evaluate the corrosion state of steel embedded in cementitious composites. Also, the phase characterizations and microstructure of the behavior of mixes containing TiO_2_ and ZnO nanoparticles were investigated using Energy Dispersive X-ray spectroscopy (EDX) and a scanning electron microscope (SEM).

## 2. Materials and Methods

### 2.1. Materials

#### 2.1.1. Cement

The cement used for this study, ordinary Portland cement (OPC) of grade 42.5, was provided by Wadi El-Nile Cement Company, Beni-Suef, Egypt, and met the specifications of ASTM C150 [32]. The initial and final setting times are 65 and 200 min, respectively. The expansion is 1 mm, and the fineness is 4200 cm^2^/gm. Table 1 shows its chemical composition.

#### 2.1.2. Aggregate

Table 2 lists the physical characteristics of the sand and crushed dolomite utilized in this study. The tests were carried out in accordance with BS 882’s guidelines (1983). Table 3 summarizes the chemical composition of aggregates.

#### 2.1.3. Nano-Materials

Nano-materials were prepared using a chemical method at the Faculty of Postgraduate Studies for Advanced Sciences (PSAS) at Beni-Suef University, Beni Suef, Egypt. These agree well with earlier research [8,33]. Using the Scherrer equation (Equation (8)), the average particle size of the used nano-materials was determined.

(8) $$D=\frac{K\lambda }{\beta \cos\theta }$$

where ***D*** represents average particle size, ***K*** represents the Scherrer constant (0.9), ***λ*** represents X-ray wavelength, ***β*** represents the full width at half maximum of the sharp peaks (FWHM), and ***θ*** represents the diffraction angle (radians).

##### TiO_2_ (Titanium Dioxide)

**Figure 1:**
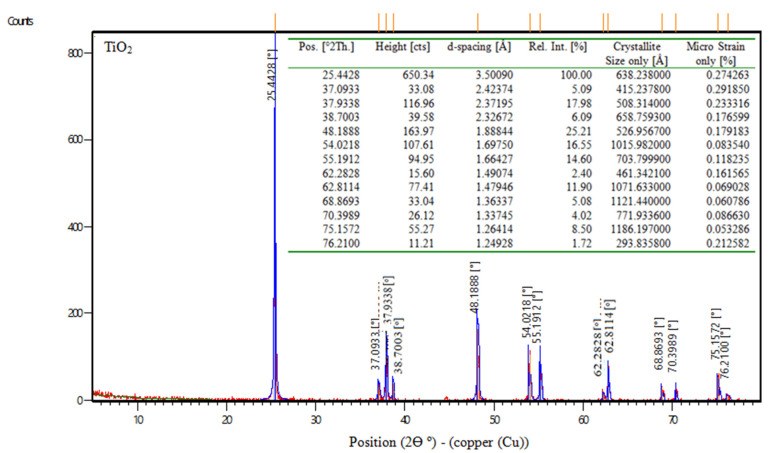
XRD data of nps-TiO_2_.

TiO_2_ is the chemical formula for titanium dioxide, often known as titania, which is a naturally occurring form of titanium oxide. According to the manufacturer, the physical characteristics of titanium dioxide are displayed in Table 4. Also, Figure 1 shows the XRD of nps-TiO_2_.

##### ZnO (Zinc Oxide)

**Figure 2:**
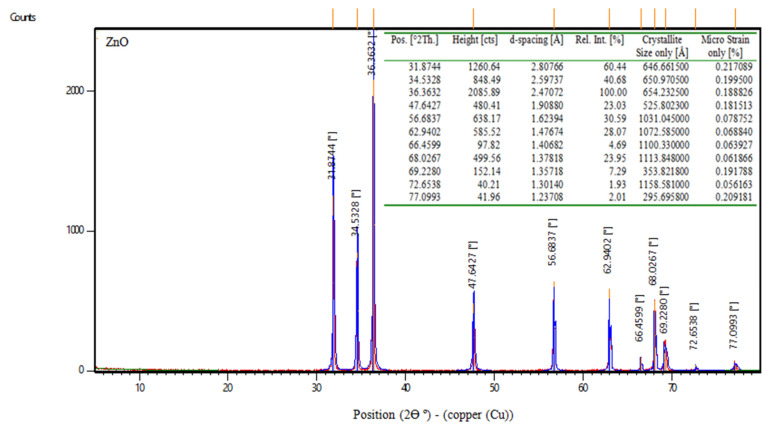
XRD data of nps-ZnO.

Zinc oxide, abbreviated ZnO, is an inorganic compound. ZnO is a white powder that is water insoluble. According to the manufacturer, the properties of zinc oxide are shown in Table 5. Moreover, Figure 2 displays the XRD of nps-ZnO.

#### 2.1.4. Water

In this study, fresh tap water and salt water were used.

- **For fresh tap water**: the samples were cast in clean, impurity-free (lacking impurities, such as salts, organic compounds, acids, oils, etc.) fresh tap water, which was also employed in the fresh tap-water-curing series. In accordance with ASTM D 1193 [34], it was also devoid of silt, clay, and other elements that could have harmed the steel-reinforced concrete or other materials. The water used for mixing, nevertheless, had a pH of 7.
- **For salt water**: water for the series of seawater cures came from Qaroun Lake in Al-Fayoum, Egypt. Table 6 contains a list of this water’s chemical elements.

#### 2.1.5. Super-Plasticizers

In this study, the super-plasticizer Sikament^®^-NN, which has a density of 1.2 at 20 °C (its properties are shown in Table 7), is added to the fresh concrete to improve its workability. It complies with ASTM C494 [35] specifications (Type F). The ideal ratio for the super-plasticizer to improve the workability of the mixture without lowering its mechanical strength is 1% of the weight of the cement.

#### 2.1.6. Phenolphthalein Solution (Phph Dye)

Phenolphthalein Solution (Phph Dye) is both a chemical substance and a dye. It is used for the determination of photocatalytic activity. In this study, the phenolphthalein detector and manual (Phph) were used. In order to obtain a 1000 ppm concentration, 0.125 g of phenolphthalein were added to 75 mL of sodium hydroxide and supplemented with 50 mL of distilled water. A total of 0.5 g of phenolphthalein was added to 75 mL of sodium hydroxide, which was then diluted with 50 mL of distilled water to achieve 4000 ppm.

### 2.2. Concrete Mix Design

#### 2.2.1. Features of Concrete Mixtures

In this study, three series were used. The first one was the concrete cubes (100 mm) used for the compressive strength test, the second was the prismatic specimens (40 mm × 40 mm × 160 mm) used for the cement mortar to evaluate self-cleaning tests, and the third was the cylindrical specimens with a diameter of 70 mm and a length of 100 mm used for the corrosion test. The control mix in this research was created to provide 30 N/mm^2^ of compressive strength after 28 days of curing, with a water-to-binder/cement ratio (w/c) of 0.45 for the used mixes. The mix’s proportions are listed in Table 8 for reference. This mixture produced compressive strengths of 28.9 and 44.5 N/mm^2^ for 7 and 28 days, respectively.

#### 2.2.2. Preparing Mixes

- First, all the components were weighed: the cement, the dolomite, the sand, and finally the water, including the superplasticizer and nano-materials for the TiO_2_ and ZnO specimens.
- For the concrete mixes of cubes and cylinders, mixing was performed in the mixer. Firstly, the dry components—dolomite, cement, and sand—were mixed for one minute before adding another ingredient. Then, water was added, including the superplasticizer (with or without nano-materials), and was mixed using a stirrer. The mixing process should take at least two minutes after adding the dry components, and one minute after adding the water.
- For the mortar mixes of prismatic specimens, mixing was performed by hand. Firstly, water and nps-TiO_2_ or nps-ZnO were mixed together by using a stirrer for a further 5–8 min to provide a uniform distribution. Then, the superplasticizer was added to them during the mixing. Thereafter, cement and sand were introduced. Then, the previous mixture was added to the cement and sand, and they were mixed for a further 5 min to obtain a homogeneous mix.

## 3. Experimental Plan

### 3.1. Compressive and Flexural Strengths

In this study, the compressive strengths were measured using 100 mm cubes. Prisms of (40 mm × 40 mm × 160 mm) were used to evaluate the compressive and flexural strengths. The vibrating table and vibrating machine were used to compact the slides after filling them with the mortar mixture. All of the control and nps-TiO_2_ samples were demolded after 24 h and then cured for 7, 28, and 90 days in the required water; however, the nps-ZnO samples were demolded after (7–14) days, depending on the weather, and then cured for 7, 28, and 90 days in the required water in accordance with ASTM C 192 [36]. The samples were removed from the curing water a day before the test. First, by using the testing machine, the samples were loaded gradually until they fractured, and only for specimens whose breakage took place in the middle of the slide was the load of the crack recorded. The flexural strength of the concrete in N/mm^2^ was calculated from the Equation (1). Following that, the specimens were consistently loaded using the compressive machine, and the load was registered when failure occurred in half of the slide. The compressive strength (N/mm^2^) is obtained by dividing the rupture load (N) by the cross-sectional area (mm^2^).

(9) $$\sigma_{f}=\frac{PL}{AB^{2}}$$

where (***σ_f_***) expresses the flexural strength of the concrete in N/mm^2^ and ***P*** is the fracture load in Newton. In addition to ***A***, ***B***, and ***L*** are the width, depth, and length of the beam, respectively, and they are all measured in mm.

### 3.2. Corrosion Rate

**Figure 3:**
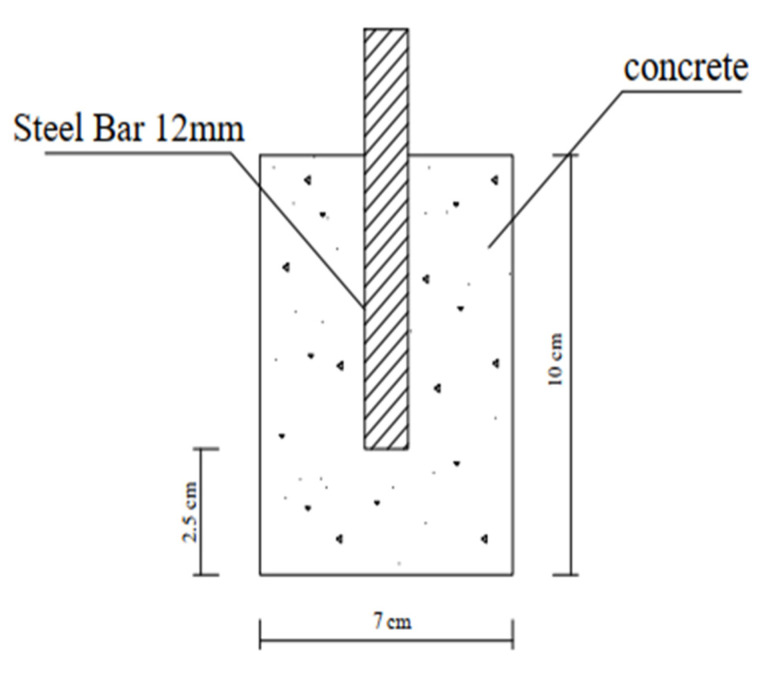
Schematic diagram of the cylindrical sample.

The corrosion rate was determined using cylindrical specimens, as shown Figure 3 (diameter of 70 mm and length of 100 mm) with a 12 mm diameter and a 100 mm long rebar in their middle. The cylinders were cast using the mix, with the rebar positioned vertically, in the center, and parallel to the long side of the mold, where Figure 3 shows the position of the rebar in the middle and parallel to the length of the cylinder. Layers of the mix were filled in the mold, and after being equally distributed, the mold was compacted in the vibrating table. After 24 h, the control and nps-TiO_2_ samples were demolded and allowed to cure for six months in the determined water (fresh or salt water), but nps-ZnO samples were demolded after (10–14) days and also cured in the determined water (fresh or sea water) for six months. Potentiostat was used to measure corrosion parameters (electronic hardware to control the 3-electrode-cell that runs the electro-analytical experiments). To obtain the corrosion rate, a linear polarization resistance (LPR) (5.1.0) test was used.

#### Linear Polarization Resistance (LPR)

**Figure 4:**
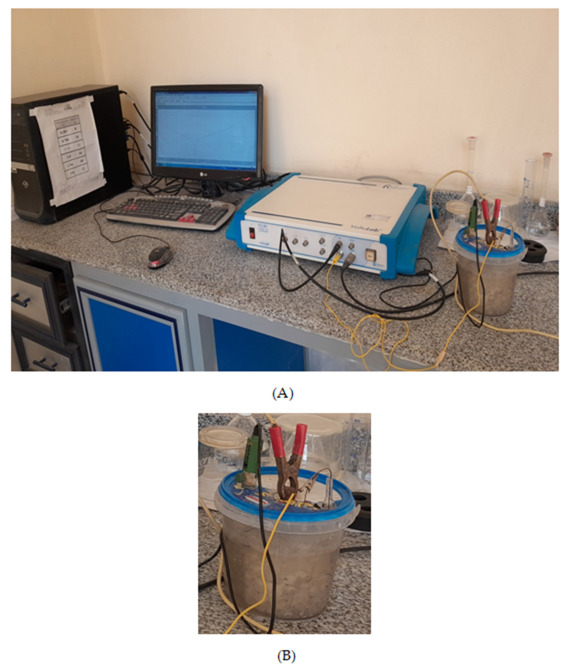
Corrosion potential testing set-up: (**A**) corrosion potential testing setup during test; (**B**) the tested sample submersed in fresh tap water.

The LPR test is one of the non-destructive tests used to determine corrosion rate because it is a simple and easily applicable test. It is obtained through a potentiostat connected to a computer running the software VOLTAMASTER 4, and a corrosion container with a three-part electrode (the reference electrode, the working electrode, and the counter electrode). Before the test, the tested cylinders must spend 10–15 min submerged in the water to make sure the specimens are fully saturated, which is necessary to determine the corrosion rate accurately. The potentiodynamic polarization set-up is shown in Figure 4.

### 3.3. Phenolphthalein Discoloration Test

**Figure 5:**
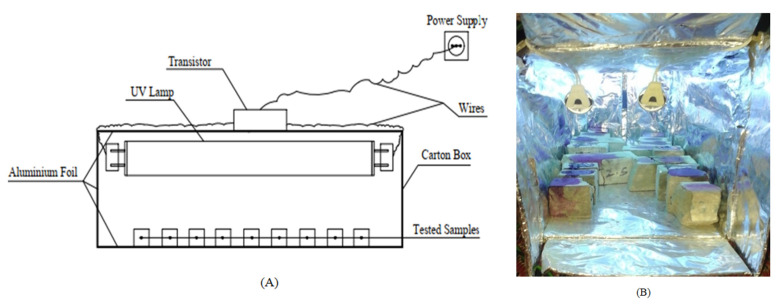
Schematic diagram for UV light set-up: (**A**) the apparatus and (**B**) the apparatus during the test.

In this test, the concrete’s ability to self-clean is determined by the exposure of samples to sunlight or UV light. The Phenolphthalein solution (Phph) is applied on surfaces subjected to irradiation under sunlight or UV light. On the surface of the casted concrete cubes, 10 mL of Phph dye is dropped on each sample and placed under direct sunlight or UV light. The discoloration of Phph under natural sunlight takes up to 3 h after irradiation has been conducted. After 1 h and 3 h, the samples were observed visually to find the degradation of (Phph) on the concrete surfaces. To test the samples under UV light, a simple apparatus was prepared, as shown in Figure 5.

### 3.4. Instrumentation

#### 3.4.1. Scanning Electron Microscope (SEM)

The microstructure of self-cleaning concrete samples is identified through SEM analysis. The shape, content, and crystallography of the specimen microstructure are clarified and described using SEM technology. The crushed chips from the studied samples in this study were gathered for microscopic characterization. Since concrete is regarded as a conductive material, the concrete chips were coated with gold to stop the concrete molecules from charging with electrons. The use of flat and thin chips allowed for the collection of images with great quality, contrast, and brightness while avoiding charging any surface flaws.

#### 3.4.2. Energy Dispersive X-ray Spectroscopy (EDX)

EDX is one analytical method for chemical or analytical characterizing materials. The EDX is utilized as a supplement to the semi quantitative analysis. EDX analysis yields the **element proportion** or **atomic proportion** of each element as a result. A spectrum produced by EDX analysis shows the peaks associated with the sample under the investigation’s elemental makeup. This characterization technique can also be used to construct a sample’s elemental mapping. The EDX study used the identical chips that were chosen for the SEM investigation.

## 4. Results and Discussion

### 4.1. Compressive Strength Test

**Figure 6:**
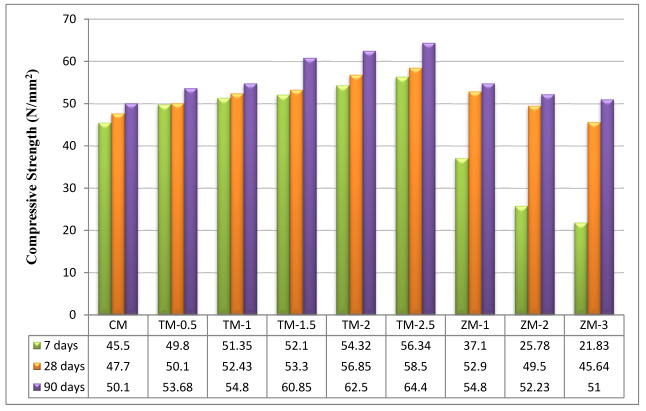
The specimens’ compressive strengths after 7, 28, and 90 days of curing in fresh tap water.

The values of the control and the nano-material specimens’ compressive strengths after 7, 28, and 90 days of curing in fresh tap water are listed in Figure 6. Figure 6 compares the compressive behaviors of fresh tap-water-cured nps-TiO_2_ mixes (TM-0.5, TM-1, TM-1.5, TM-2, and TM-2.5), and nps-ZnO mixes (ZM-1, ZM-2, and ZM-3) to the control mix (CM). The strengths of the fresh tap-water-cured control mix were used as a guide for the corresponding curing intervals. According to the results, the compressive strength for nps-TiO_2_ mixes improved with increasing nps-TiO_2_ concentrations (as shown in Figure 6), and the optimum values were recorded with 2.5% of nps-TiO_2_. The increment ratios of the sample containing 2.5% of nps-TiO_2_ are 23.82%, 22.64%, and 28.54% at 7, 28, and 90 days, respectively. The increase in compressive strength with 2.5% of nps-TiO_2_ is attributed to nps-TiO_2_’s non-reactive fine filler properties, which reduced porosity, lacked pozzolanic activity, and played a significant role in ensuring cement matrix homogeneity [22,37,38,39,40,41]. Nps-TiO_2_ can also speed the formation of C-S-H, lower CH crystallization, and raise the hydration degree in the early stages [42]. Nevertheless, the optimal nps-ZnO percentage for samples was 1%, as increasing nps-ZnO percentages decreased compressive strength compared with ZM-1. These results are consistent with those reported by Nayak et al. [43], Liu et al. [42], and Thangapandi et al. [44]. ZM-1 increased the corresponding strengths by 10.90% and 9.38% at 28 and 90 days, respectively. But the percentage was −18.46% at 7 days. In light of this analysis, it is important to note that the compressive strength results for nps-ZnO specimens at 7 days were lower than those for the control mix. This is because nps-ZnO has an impact on the strength and setting time of concrete, particularly at early stages because it decreases the cement’s degree of hydration. These findings support what was noted by Liu et al. [42], Nayak et al. [43], Kumar et al. [45], and Garg et al. [46], which may be linked to the reaction between nps-ZnO and CH, per Equations (9)–(12) [43]. Consequently, at 7 days, the cement mortar containing nps-ZnO particles had a lower degree of hydration than the control. This fact may be connected to the following interaction between Ca(OH)_2_ and nps-ZnO [47], as shown in Equation (12). This reaction creates a layer on the surface, which restricts the transport of water to C3S and slows the setting time. Moreover, due to the filler action of nps-ZnO [42,47,48] at 28 days, the improvement of compressive strength was significant.

(10) $$2\left(3CaO\cdot SiO_{2}\right)+6H_{2}O\to 3CaO\cdot 2SiO_{2}\cdot 3H_{2}O+3Ca{\left(OH\right)}_{2}$$

(11) $$Ca{\left(OH\right)}_{2}+ZnO\to CaO+Zn{\left(OH\right)}_{2}$$

(12) $$Zn{\left(OH\right)}_{2}+2HCl\to Zn{Cl}_{2}+2H_{2}O$$

(13) $$2ZnO+Ca{\left(OH\right)}_{2}+4H_{2}O\to Ca{\left(Zn{\left(OH\right)}_{3}H_{2}O\right)}_{2}$$

### 4.2. Flexural Strength

**Figure 7:**
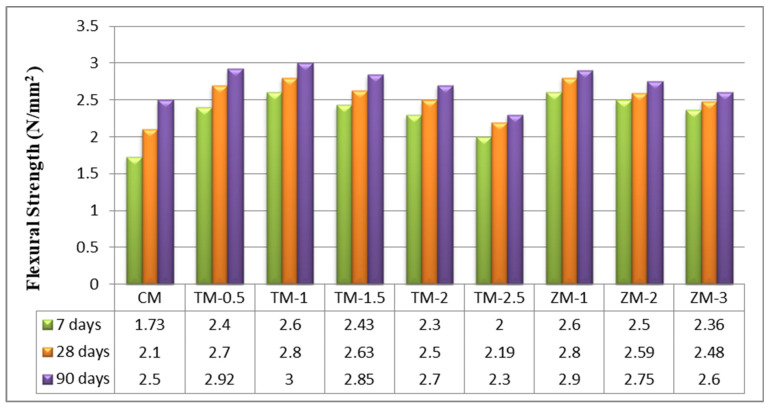
The specimens’ flexural strengths after 7, 28, and 90 days of curing in fresh tap water.

The values of the control and nano-specimens’ flexural strengths after 7, 28, and 90 days of curing in fresh tap water are listed in Figure 7. According to the current results, the optimal value for nps-TiO_2_ is TM-1 (1%). The increasing percentages for this sample were about 33.46%, 33.33%, and 20% for 7, 28, and 90 days, respectively. The improved flexural strength may be due to the enhanced C-S-H gel formation that occurs in the presence of TiO_2_ nanoparticles. This is the same as what was researched by Nazari et al. [49]. Furthermore, because nps-TiO_2_ is a stable, non-reactive fine filler [22], it does not form a network capable of withstanding tensile pressures produced by the flexural load. For nps-ZnO, the results of this research show that the higher the nps-ZnO ratio the lower the flexural strength, and this is the same information that was investigated by Kumar et al. [45]. The increasing percentages for a sample containing 1% of nps-ZnO (the optimum ratio for samples of nps-ZnO) are 50.29%, 33.33%, and 16% at 7, 28, and 90 days, respectively.

### 4.3. Corrosion Rate

**Figure 8:**
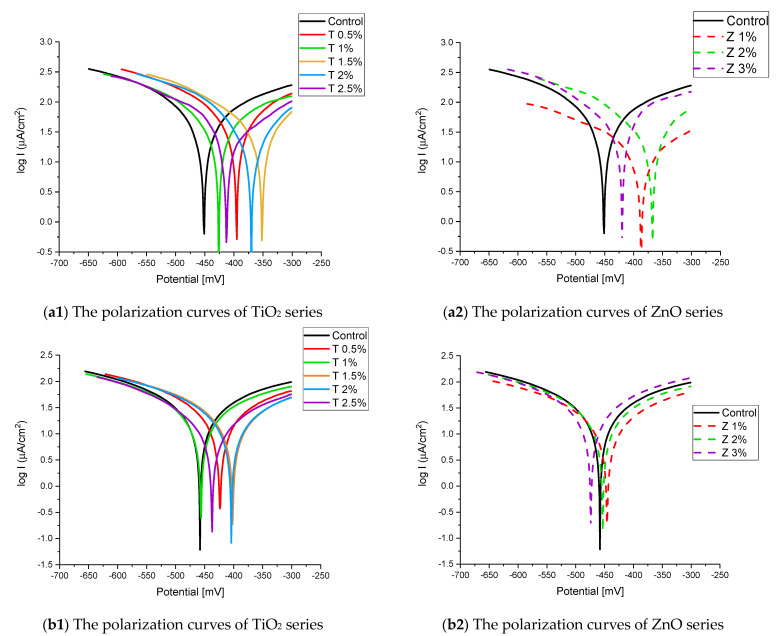
The polarization curves of the tap-water-cured series: (**a**) after 2 months and (**b**) after 6 months.

**Figure 9:**
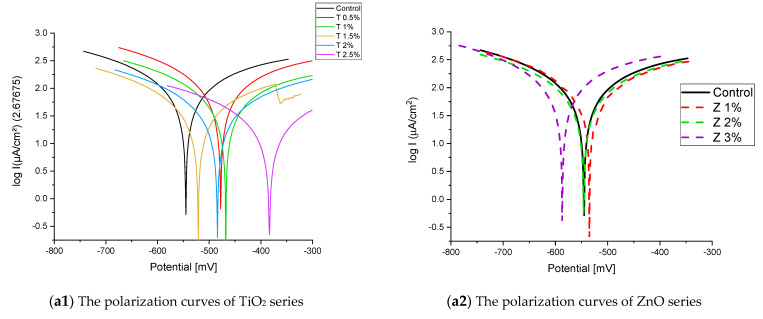

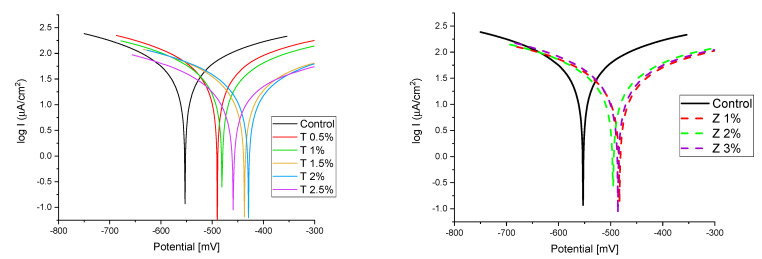
The polarization curves of the Qaroun’s Lake water-cured series: (**a**) after 2 months and (**b**) after 6 months.

**Figure 10:**
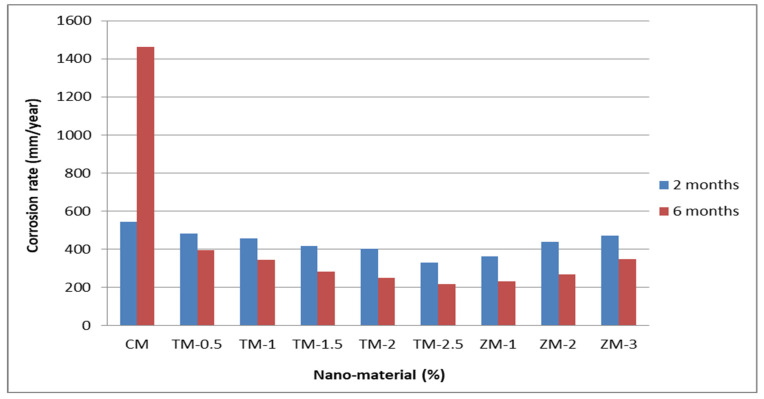
Corrosion rate in rebar bars of specimens that were cured in fresh tap water.

**Figure 11:**
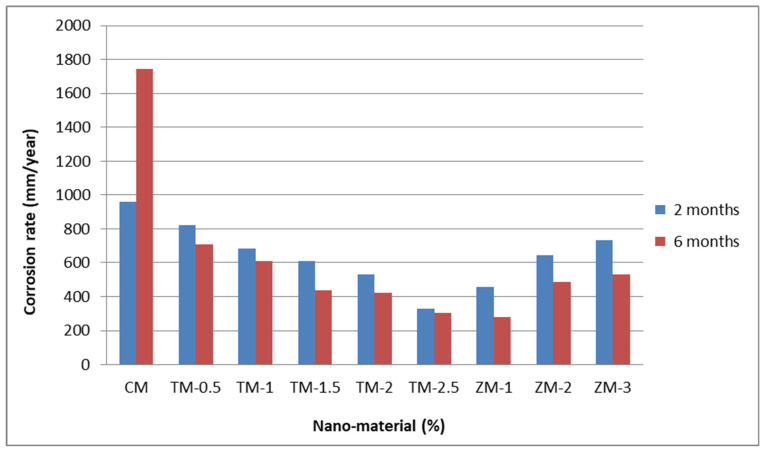
Corrosion rate in rebar bars of specimens that were cured in Qaroun’s Lake water.

Figure 8 and Figure 9 show the Tafel potentiodynamic polarization curves for each specimen exposed to fresh tap water and salt water, respectively, after 2 and 6 months. For every mix, half of the samples were cured in fresh tap water (normal water) and the other half in water from Qaroun Lake (salt water). Previous research has shown that seawater negatively affects and damages concrete [50,51], and this study’s findings are in line with previous studies’ findings, as shown in Table 9, Figure 10, Table 10, and Figure 11. For nps-TiO_2_ specimens, according to this research, it can be noted that the corrosion rate gradually decreases with the increasing nps-TiO_2_ ratio. This may occur because nps-TiO_2_ increases the amount of C–S–H gel and decreases amount of pores [22,39,41]. On the other hand, for nps-ZnO samples, the results show that increasing the nps-ZnO ratio about 1% increases the corrosion rate (these results agreed with those noted by Thangapandi et al. [44]). This could be due to the fact that nps-ZnO delays the setting time [42,45]. The results of the inhibitor efficiency (ƞ) show that nps-ZnO samples have higher efficiency than nps-TiO_2_ samples, as illustrated in Table 11, Table 12, Table 13 and Table 14.

### 4.4. Results of Phenolphthalein Discoloration Test

**Figure 12:**
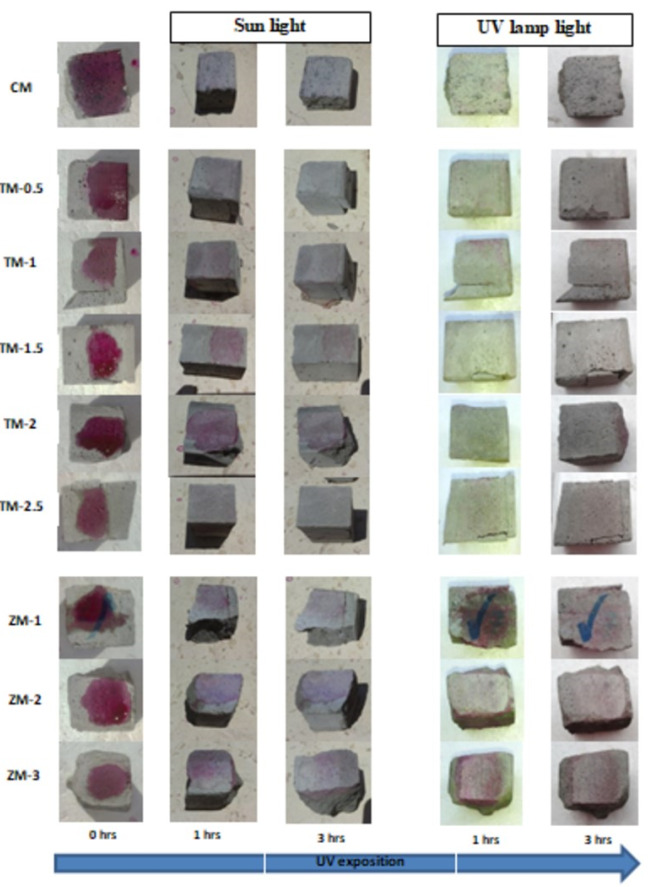
Phenolphthalein discoloration based on the duration of sun light and UV exposure for the specimens at 7 days of curing using 1000 ppm concentration of solution.

**Figure 13:**
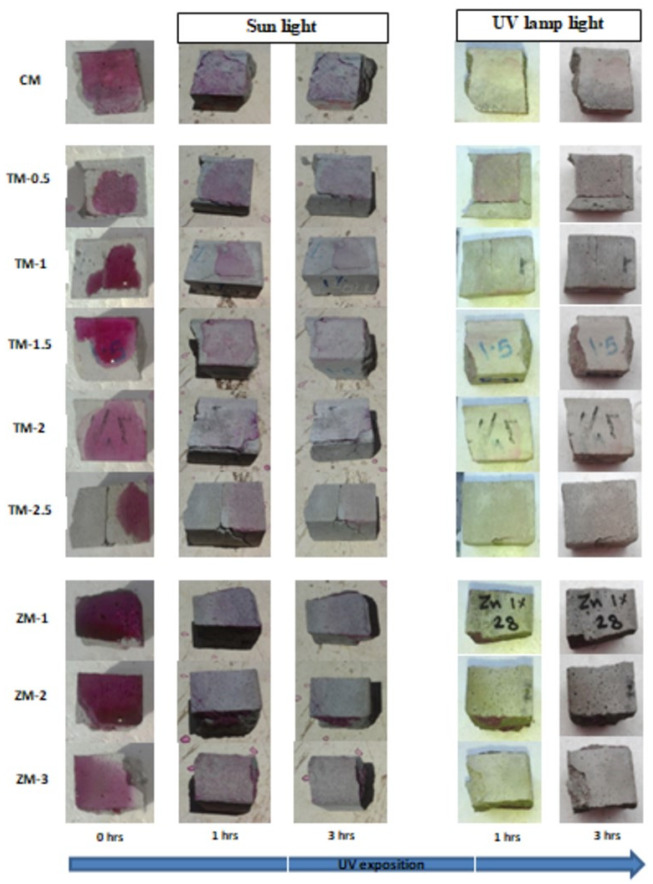
Phenolphthalein discoloration based on the duration of sun light and UV exposure for the specimens at 28 days of curing using 1000 ppm concentration of solution.

**Figure 14:**
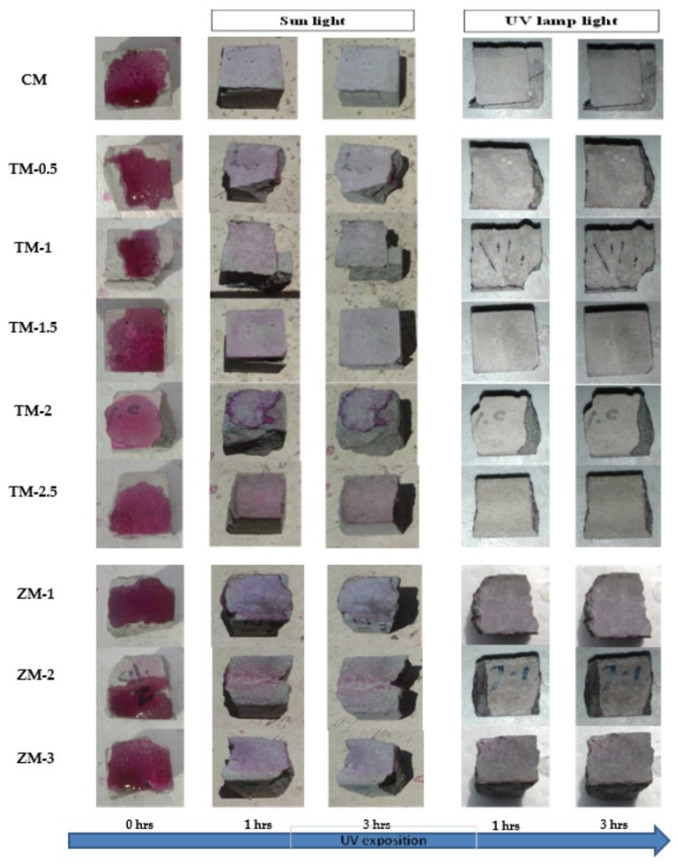
Phenolphthalein discoloration based on the duration of sun light and UV exposure for the specimens at 90 days of curing using 1000 ppm concentration of solution.

**Figure 15:**
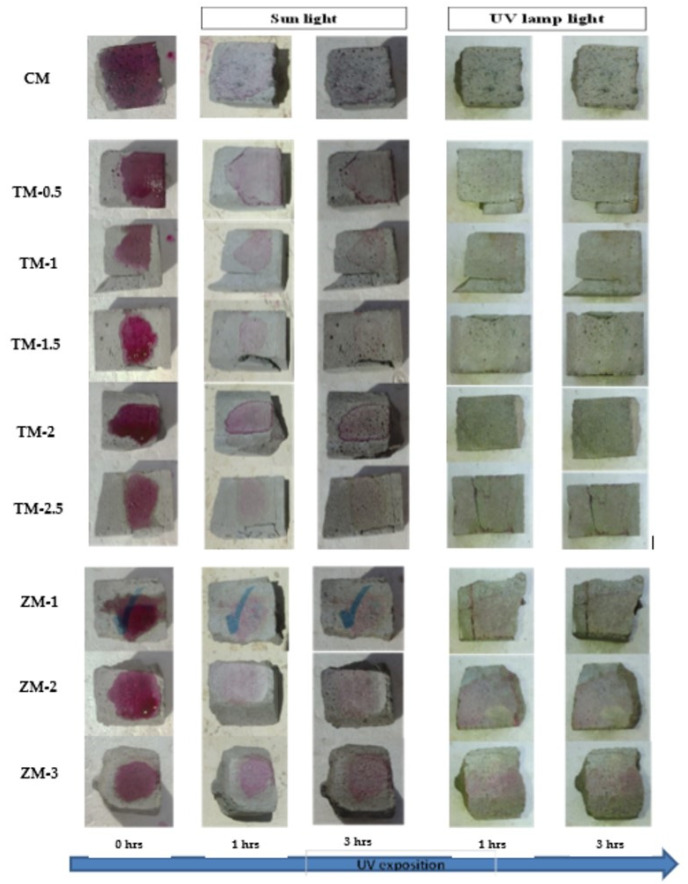
Phenolphthalein discoloration based on the duration of sun light and UV exposure for the specimens at 7 days of curing using 4000 ppm concentration of solution.

**Figure 16:**
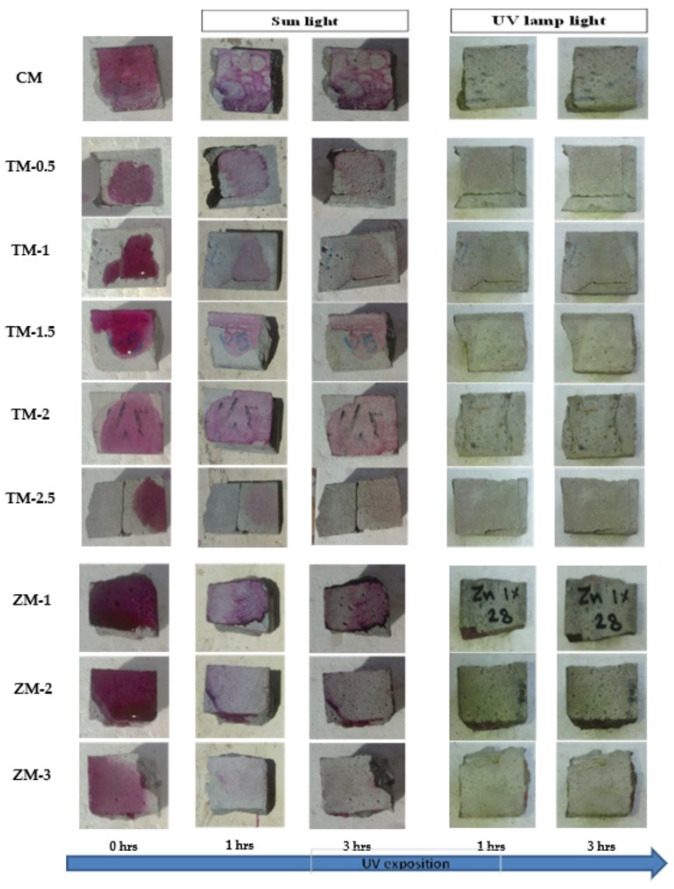
Phenolphthalein discoloration based on the duration of sun light and UV exposure for the specimens at 28 days of curing using 4000 ppm concentration of solution.

**Figure 17:**
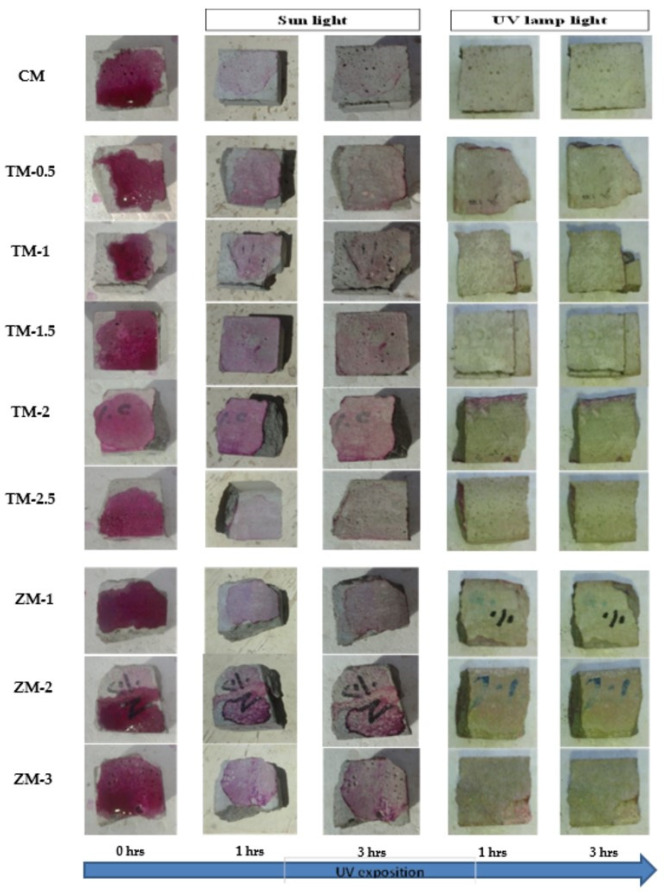
Phenolphthalein discoloration based on the duration of sun light and UV exposure for the specimens at 90 days of curing using 4000 ppm concentration of solution.

Phenolphthalein Discoloration is a different approach that has been used to assess photocatalytic activity [52]. Depending on the duration of ultraviolet (UV) exposure, specimens colored with Phenolphthalein dye are shown in Figure 12, Figure 13 and Figure 14 at 1000 ppm concentration of solution and Figure 15, Figure 16 and Figure 17 at 4000 ppm concentration of solution. It is evident that as exposure time is increased, the samples are gradually self-cleaning because of photocatalytic degradation. The results show that nps-TiO_2_ samples give better results in general than nps-ZnO samples, like the one described by Amor et al. [52], but these results are in contrast with those reported by K. Loh et al. [53]. For nps-TiO_2_ samples, by increasing the titanium content, the sample’s ability to self-clean improves; this result is similar to the argument made by Amor et al. [52]. For UV-lamp light, the results of the samples are much better compared to the effect of sunlight, but titanium samples are still better than zinc samples. Noting all the results, we find that the TM-2.5% sample always gives the best result in all stages, even with the increased concentration of the solution.

### 4.5. Microstructure Analysis

**Figure 18:**
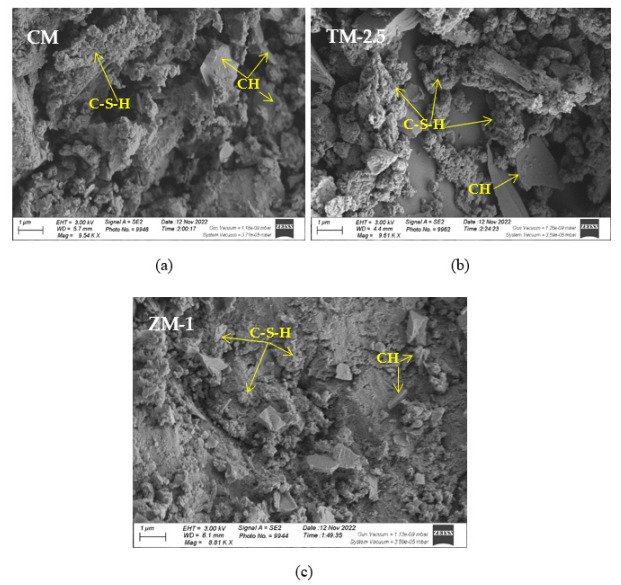
SEM micrographs of (**a**) control mix, (**b**) nps-TiO_2_, and (**c**) nps-ZnO specimens at 90 days of curing age.

**Figure 19:**
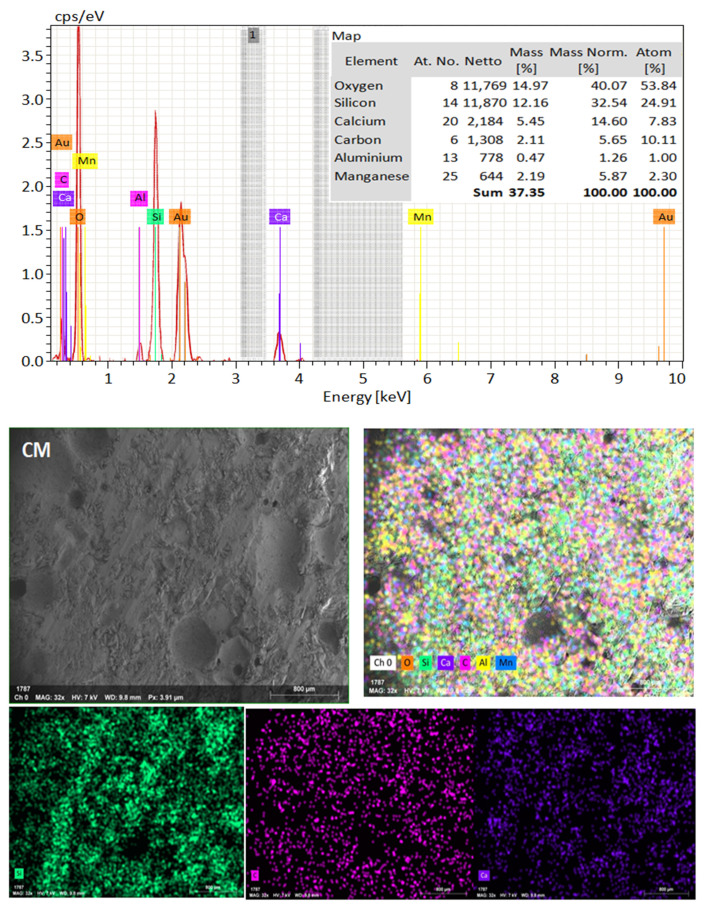
EDX and Mapping analysis for control mix at 90 days of curing age.

**Figure 20:**
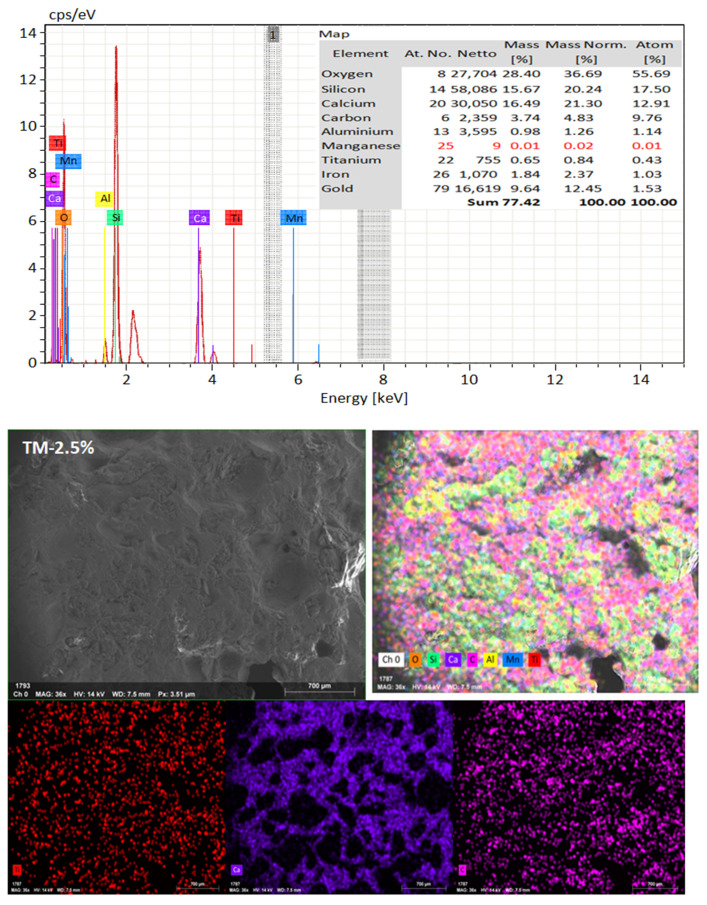
EDX and Mapping analysis for nps-TiO_2_ sample at 90 days of curing age.

**Figure 21:**
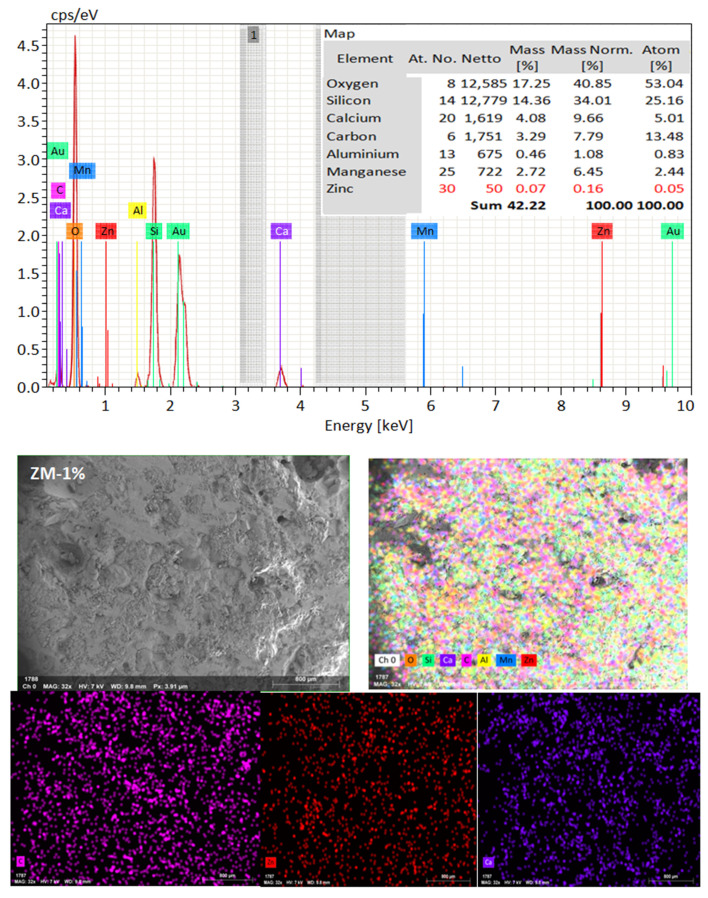
EDX and Mapping analysis for nps-ZnO sample at 90 days of curing age.

SEM frequently does in-depth microstructure examination, as illustrated in Figure 18, which displays the SEM images of the control, TM-2.5%, and ZM-1% samples at 90 days. For the control sample, there are many large-sized pores. Conversely, nano-samples have fewer pores compared to the control sample. Furthermore, the nps-TM specimen has low levels of calcium hydroxide (CH) and significant amounts of calcium silicate hydrate (C-S-H) that are absent from the control image. Figure 19, Figure 20 and Figure 21 show, respectively, the mapping images for specimens of the control, TM-2.5%, and ZM-1% that were treated in fresh tap water for 90 days and their corresponding EDX spectra. Also, Figure 19, Figure 20 and Figure 21 include the tables that list the expected chemical compounds of these specimens as well as the associated individual chemical components. According to these results, notice that the percentage of calcium in the nps-ZnO mixture is lower than that of the control combination, demonstrating the delay in setting time.

## 5. Conclusions

Based on our results, it can be concluded that adding the photocatalyst oxide powder of nano-materials (TiO_2_ and ZnO) to Portland cement mortar improves its mechanical, electrochemical, and photocatalytic characteristics. Furthermore, this effect is valid for all cement-based materials. The study’s findings can be summarized as follows:

- In nps-TiO_2_ and nps-ZnO mixes, the best percentages for compressive strength are TM-2.5% and ZM-1%. However, the optimal ratios for flexural strength are 1% for nps-TiO_2_ mixtures and 1% for nps-ZnO mixtures. With higher nps-TiO_2_ concentrations, the nps-TiO_2_ mixture’s compressive strength increased, with 2.5% of nps-TiO_2_ being the ideal concentration. As a result, at 7, 28, and 90 days, the increment ratios of the sample containing 2.5% of nps-TiO_2_ were 23.82%, 22.64%, and 28.54%, respectively.
- As increasing nps-ZnO percentages reduce compressive strength in comparison to ZM-1, the ideal nps-ZnO percentage for samples is 1%. At 28 and 90 days, ZM-1 improved the corresponding strengths by 10.90% and 9.38%, respectively.
- For the flexural strength test, TM-1 (1%), for nps-TiO_2_, is the ideal value. For this sample, the percentages increased by about 33.46%, 33.33%, and 20% for 7, 28, and 90 days, respectively. Additionally, the findings of this study indicate that the flexural strength of nps-ZnO samples decreases with increasing nps-ZnO ratios. For samples containing 1% nps-ZnO, the percentages rose by 50.29%, 33.33%, and 16% at 7, 28, and 90 days, respectively (this is the ideal ratio for nps-ZnO samples).
- In regard to corrosion rate, this study shows that the corrosion rate for nps-TiO_2_ specimens gradually reduces with an increasing nps-TiO_2_ percentage in all exposure conditions. This may happen as a result of increasing the volume of C-S-H gel and reducing the number of pores. However, the results for the nps-ZnO samples indicate that the corrosion rate increases when the nps-ZnO ratio increases more than 1%. Additionally, ZM-1% from the nps-ZnO mixes was the optimum percentage. It is obvious that the samples increasingly self-clean as exposure duration is extended due to photocatalytic deterioration. The findings demonstrate that nps-TiO_2_ samples perform better overall than nps-ZnO samples.
- According to this study’s findings, all samples display some degree of self-cleaning effectiveness in exposure to UV light or sunlight, even when no nano-materials are present (0%). In general, the self-cleaning effectiveness of the mixtures improves as the TiO_2_ concentration rises from 0.5% to 2.5%. Although an increase in the quantity of active TiO_2_ does not considerably speed up the samples’ degradation of RhB, an increase in the TiO_2_ content is predicted to increase the amount of activated TiO_2_. Due to titanium dioxide’s photocatalytic activity, the organic material decomposes, which causes the color shift.
- Similar to the photocatalyst of nps-TiO_2_, nps-ZnO, as a nano-photocatalyst, has significant potential for use as a self-cleaning agent in concrete buildings. According to the results of this study, nps-ZnO has reduced photodegradation efficiency, this may be why it does not perform as well in these interactions. Also, the photocatalytic activity of nps-ZnO may be affected by crystallinity.

## Figures and Tables

**Table 1:** Chemical composition of cement.

Material	Fe_2_O_3_	SO_3_	SiO_2_	MgO	K_2_O	CaO	Al_2_O_3_	Na_2_O	LOI	F.L
wt%	3.22	2.39	21.20	0.69	0.50	63.41	5.50	0.10	2.30	2.70

LOI: loss on ignition; F.L: free lime.

**Table 2:** Mechanical properties of aggregates.

Property	Sand	Crushed Dolomite	Specification Limits
Fineness modulus	2.35	2.20	2–2.73
Volume weight (t/m^3^)	1.65	1.45	1.4–1.7
Specific gravity (t/m^3^)	2.5	2.55	2.5–2.7

**Table 3:** Chemical composition of aggregates.

Material	Chemical Composition (%)										
	CaO	K_2_O	MgO	SiO_2_	Al_2_O_3_	Na_2_O	MnO	SO_3_	P_2_O_5_	LOI	Cl
Sand	0.47	0.64	0.31	91.40	3.32	0.63	0.02	0.24	0.06	1.13	0.14
Crushed dolomite	32.01	0.01	19.18	1.74	0.05	0.11	–	–	–	46.20	–

**Table 4:** Properties of TiO_2_.

Property	Results
Average particle size (nm)	25 ± 5
Density (g/cm^3^)	4.1
Purity (%)	99%
Color	white
Shape	Powder

**Table 5:** Properties of ZnO.

Property	Results
Average particle size (nm)	30 ± 10
Density (g/cm^3^)	5.6
Purity (%)	99%
Color	white
Shape	Powder

**Table 6:** The chemical properties of the water in Qaroun Lake.

Property	Results
Density	1.025 gm/cm^3^
Sodium	10.109 gm/L
Sulfate	9.712 gm/L
Chlorides	12.985 gm/L
Calcium	0.500 gm/L
Bicarbonate	0.305 gm/L
Carbonates	0.030 gm/L
Magnesium	1.325 gm/L
Soluble salts	35.438 gm/L
Others	0.472 gm/L
Ions	–

**Table 7:** Technical Data for Super plasticizer Sikament^®^—NN.

**Base**	Naphthalene formaldehyde sulfonate
**Color**	Dark brown liquid
**Density (at 20 °C)**	1.20 ± 0.005 kg/lit.
**Chloride content**	Free chloride
**Compatibility**	All types of Portland cement, including sulfate resistor cement

**Table 8:** Mix design.

Specimens	Sand (kg/m^3^)	Crushed Dolomite (kg/m^3^)	Cement (kg/m^3^)	Water (kg/m^3^)	Super- Plasticizer (%)	nps-TiO_2_ (%)	nps-ZnO (%)
CM *	596.5	1108.10	300	135	1	-	-
**CM**	0.4142	-	0.1381	0.0552	1	-	-
**TM-0.5**	0.4142	-	0.1381	0.0552	1	0.5	-
**TM-1**	0.4142	-	0.1381	0.0552	1	1	-
**TM-1.5**	0.4142	-	0.1381	0.0552	1	1.5	-
**TM-2**	0.4142	-	0.1381	0.0552	1	2	-
**TM-2.5**	0.4142	-	0.1381	0.0552	1	2.5	-
**ZM-1**	0.4142	-	0.1381	0.0552	1	-	1
**ZM-2**	0.4142	-	0.1381	0.0552	1	-	2
**ZM-3**	0.4142	-	0.1381	0.0552	1	-	3

* concrete cubes (100 mm) used for the basic mix. CM: Control Mix; TM: nps-TiO_2_ Mix; ZM: nps-ZnO Mix. These mixes for casting one mortar slide (4 × 4 × 16) cm^3^.

**Table 9:** Corrosion rate at 2 and 6 months of exposure to fresh tap water.

Mix	C_rate_ (mm/Year) at 2 Months	C_rate_ (mm/Year) at 6 Months
**CM**	545	1462
**TM-0.5**	483	396
**TM-1**	458	343
**TM-1.5**	417	284
**TM-2**	401	250
**TM-2.5**	329	218
**ZM-1**	362	233
**ZM-2**	437	269
**ZM-3**	471	347

**Table 10:** Corrosion rate at 2 and 6 months of exposure to Qaroun’s Lake water.

Mix	C_rate_ (mm/Year) at 2 Months	C_rate_ (mm/Year) at 6 Months
**CM**	958	1742
**TM-0.5**	820	710
**TM-1**	685	608
**TM-1.5**	610.3	435
**TM-2**	530	420
**TM-2.5**	327	306
**ZM-1**	458	280
**ZM-2**	642	486
**ZM-3**	734	531

**Table 11:** Percentages of inhibitor efficiency at 2 months for nps-TiO_2_ specimens.

Mix	Inhibitor Efficiency (ƞ) %	
	Fresh Tap-Water-Cured Specimens	Qaroun’s Lake Water-Cured Specimens
**TM-0.5**	11.38	14.41
**TM-1**	15.96	28.50
**TM-1.5**	23.49	36.29
**TM-2**	26.42	44.68
**TM-2.5**	39.63	65.87

**Table 12:** Percentages of inhibitor efficiency at 2 months for nps-ZnO specimens.

Mix	Inhibitor Efficiency (ƞ) %	
	Fresh Tap-Water-Cured Specimens	Qaroun’s Lake Water-Cured Specimens
**ZM-1**	33.58	52.19
**ZM-2**	19.82	32.99
**ZM-3**	13.58	23.38

**Table 13:** Percentages of inhibitor efficiency at 6 months for nps-TiO_2_ specimens.

Mix	Inhibitor Efficiency (ƞ) %	
	Fresh Tap-Water-Cured Specimens	Qaroun’s Lake Water-Cured Specimens
**TM-0.5**	72.91	59.24
**TM-1**	76.54	65.10
**TM-1.5**	80.57	75.03
**TM-2**	82.90	75.89
**TM-2.5**	85.09	82.43

**Table 14:** Percentages of inhibitor efficiency at 6 months for nps-ZnO specimens.

Mix	Inhibitor Efficiency (ƞ) %	
	Fresh Tap-Water-Cured Specimens	Qaroun’s Lake Water-Cured Specimens
**ZM-1**	84.06	83.93
**ZM-2**	81.60	72.10
**ZM-3**	76.27	69.52

## Data Availability

Not applicable.
